# Necrotizing Esophagitis: A Big Squeeze?

**DOI:** 10.1093/jcag/gwab005

**Published:** 2021-03-18

**Authors:** George Rakovich, Sébastien Rolland

**Affiliations:** Section for Thoracic Surgery, Maisonneuve-Rosemont Hospital, University of Montreal, Montreal, Quebec, Canada; Section of Gastro-Enterology, Maisonneuve-Rosemont Hospital, University of Montreal, Montreal, Quebec, Canada

**Keywords:** Esophagitis, Esophageal necrosis, Gastroesophageal reflux disease

## Abstract

Necrotizing esophagitis is rare and poorly understood. The etiologies reported in what little has been published (i.e., gastroesophageal reflux exacerbated by gastric outlet obstruction and low-flow ischemia) seem somewhat simplistic and lack any direct evidence. The following paper illustrates a recent clinical case while laying out arguments supporting esophageal spasm as a possible contributing factor.

The images depict a case of necrotizing esophagitis in a 32-year-old man having ingested cocaine (Figures 1 and 2). Necrotizing esophagitis is rare and poorly understood. In elderly patients, it has been attributed to a combination of peptic injury exacerbated by gastric outlet obstruction and low flow ischemia (1), while in younger patients having ingested cocaine, it has been attributed to vasospasm (1,2). These mechanisms remain highly speculative and lack any experimental evidence. Why should reflux cause necrosis, and why should vasospasm affect a well perfused organ like the esophagus, while sparing vulnerable portions of the mesenteric circulation? (2)

**Figure 1.**
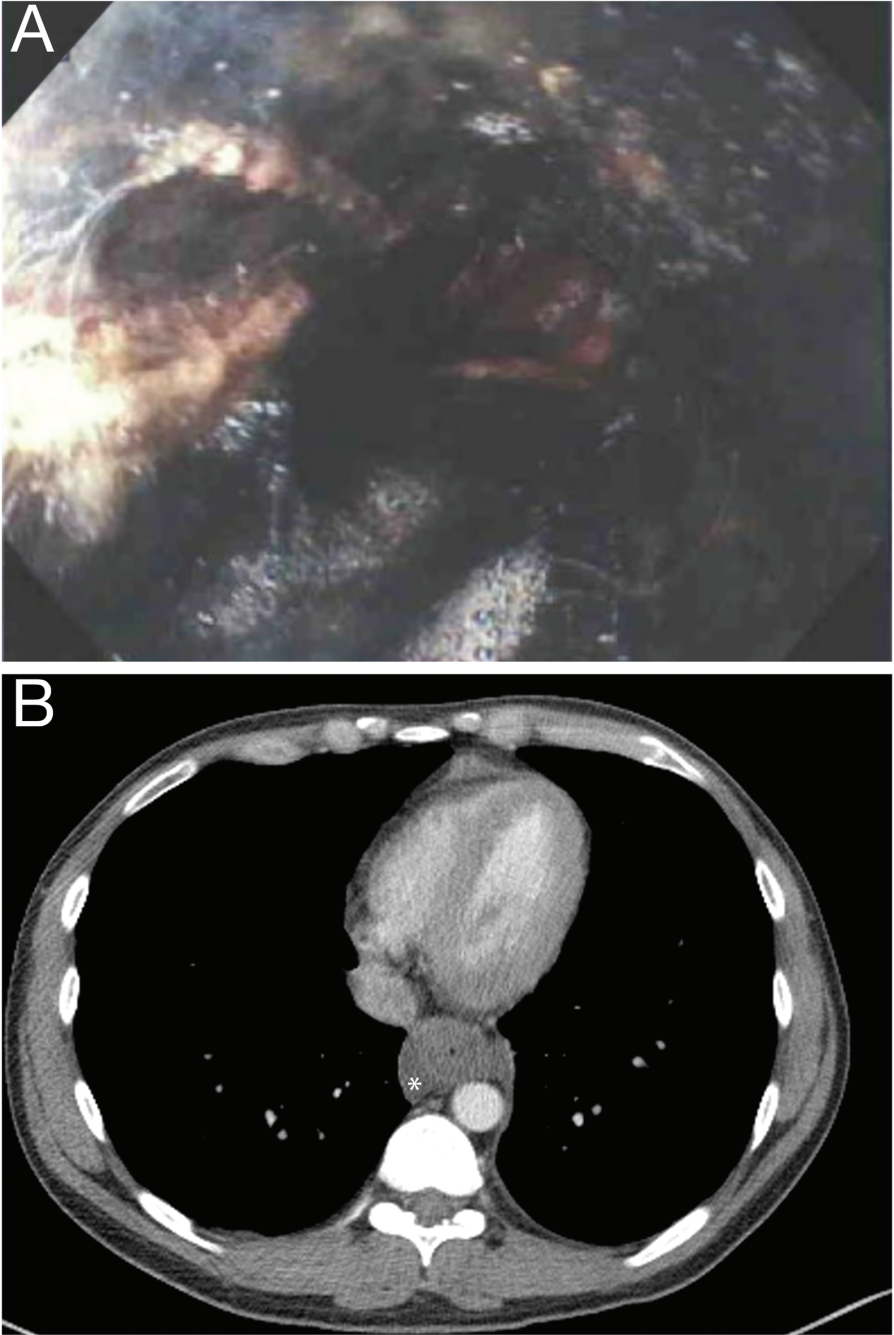
(A) Endoscopy in a patient with necrotizing esophagitis shows black mucosa. (B) CT-scan shows marked esophageal thickening and some free periesophageal fluid (asterisk).

**Figure 2.**
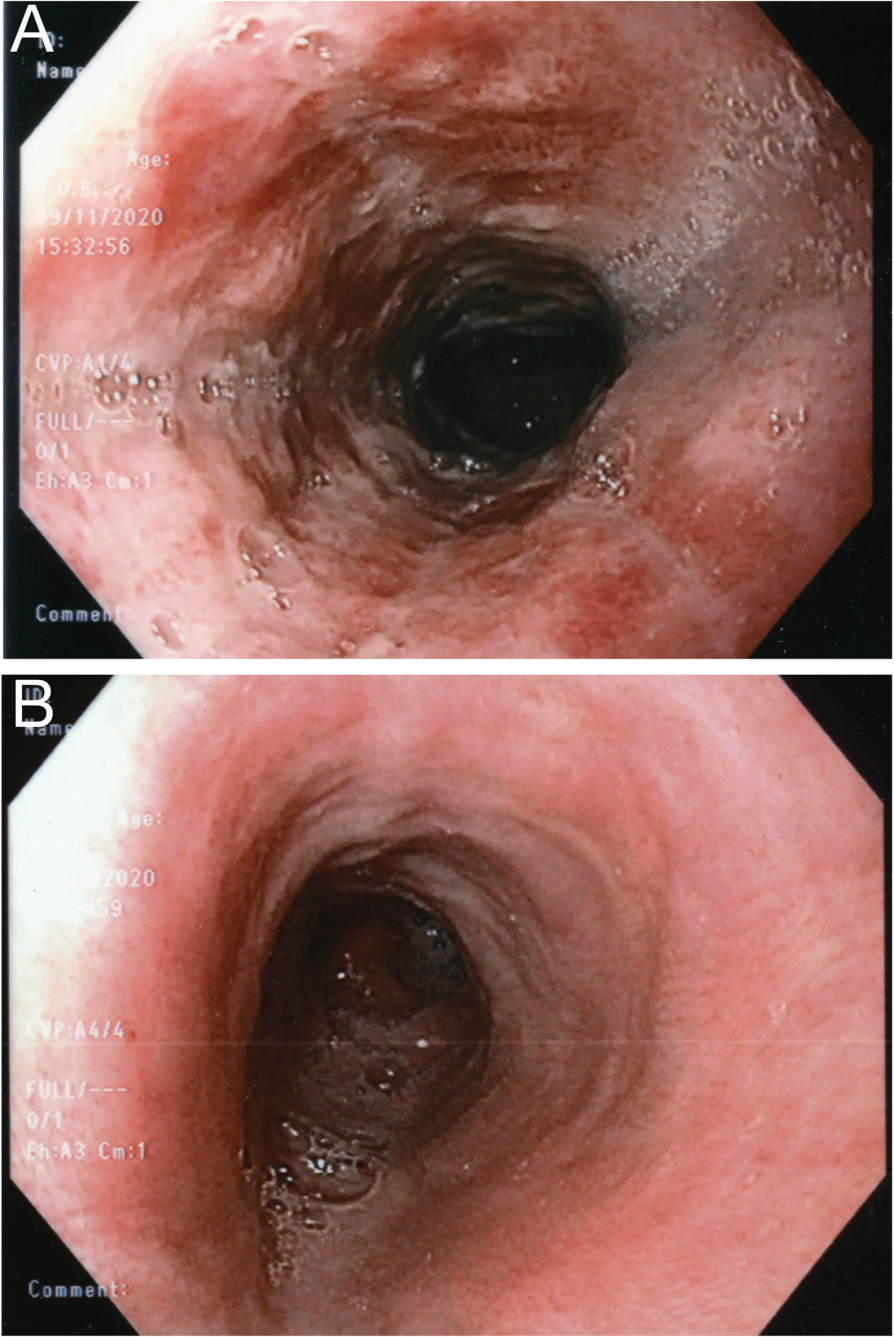
(A) Endoscopy at day 9 shows inflamed but viable mucosa in a patient having initially presented with necrotizing esophagitis. (B) At 6 weeks, the esophagus is completely healed.

On the other hand, animal studies have shown that esophageal spasm can increase luminal pressures sufficiently to cause ischemia of the esophageal wall (3). Esophageal spasm can be triggered by both local factors and neuro-humoral mechanisms (3). Esophageal spasm is a well known cause of noncardiac chest pain, and cocaine has been linked to an elusive syndrome of chest pain in the absence of documented myocardial ischemia (3,4). If one were to accept this line of reasoning, then one could reasonably argue that a severe and sustained spasm triggered by peptic reflux or cocaine-induced smooth muscle contraction could decreased parietal blood flow sufficiently to precipitate necrosis. This may be further supported by the observation that the cardial (gastric) mucosa in these cases is completely spared. Further study is required to test this hypothesis and ascertain whether treatments specifically aimed at relieving esophageal spasm may have a role in managing this condition in the future.

The authors certify that this manuscript represents original work, that they have the rights in the work, and that it is not being considered for publication elsewhere and has not already been published elsewhere.

All authors participated fully in drafting of the manuscript. All authors have read and approved submission.
